# Hypertension and Drug Adherence in the Elderly

**DOI:** 10.3389/fcvm.2020.00049

**Published:** 2020-04-07

**Authors:** Michel Burnier, Erietta Polychronopoulou, Gregoire Wuerzner

**Affiliations:** ^1^Service of Nephrology and Hypertension, Lausanne University Hospital and University of Lausanne, Lausanne, Switzerland; ^2^Hypertension Research Foundation, St-Légier, Switzerland

**Keywords:** hypertension, aging, polypharmacy, cognitive decline, depression, deprescribing

## Abstract

Hypertension is highly prevalent after the age of 65 years affecting more than 60% of individuals in developed countries. Today, there is sufficient evidence from clinical trials that treating elderly subjects with hypertension with antihypertensive medications has a positive benefit/risk ratio even in very elderly patients (>80 years). In recent years, partial or total non-adherence has been recognized as major issues in the long-term management of hypertension in all age categories. However, whether non-adherence is more frequent in hypertensive patients older than 65 years or not is still a matter of debate and the common belief is that adherence is lower in older than in younger patients. Are clinical data supporting this belief? In this brief review, we discuss the topic of drug adherence in elderly in the context of the medical treatment of hypertension. Studies show that drug adherence is actually better in patients aged 65 to 80 years when compared to younger hypertensive patients (<50 years). However, in very old patients (>80 years) the prevalence of non-adherence does increase. In this patients' group, there are specific risk factors for non-adherence such as cognitive ability, depression, and health believes, in addition to classical risk factors for non-adherence. One important aspect in the elderly is the prescription of potentially inappropriate medications that will interfere with the adherence to necessary treatments. In this context, an interesting new concept was developed few years ago, i.e., the process of deprescribing. Thus, today, in addition to conventional guidelines recommendations (use of single pill combinations, individualization of treatments), the evaluation of cognitive abilities, the regular assessment of potentially inappropriate medications, and the process of deprescribing appear to be three new additional steps to improve drug adherence in the elderly and thereby ameliorate the global management of hypertension.

## Introduction

Aging is characterized by a progressive increase of blood pressure (BP) with a steady rise of systolic BP (SBP) until the age of 70–80 years, whereas diastolic BP (DBP) increases until the age of 50–60 years and then decreases, leading to a rise in pulse pressure (1, 2). This is not a normal physiological process but rather the consequence of an age-related development of arterial stiffness and changes in arterial compliance due to our environment and life style as this age-related association has not been observed in primitive societies, suggesting that it is not a simple sequela of aging (3). The development of comorbidities such as chronic kidney disease, dyslipidemia and diabetes, which are often present simultaneously in elderly individuals, plays an important additional role in this progressive change in the BP profile occurring with age. With this age-related increase in systolic BP, it is not surprising that hypertension, defined as a SBP > 140 mmHg, is very frequent in subjects older than 60 years. Indeed, in an analysis of people aged 40–79 years who participated in 123 national health examination surveys from 1976 to 2017 in 12 high-income countries, the prevalence of hypertension was in the range of 60 to 75% in women and men older than 60 years (4). The prevalence of hypertension in older subjects has reached this high level in the beginning of the years 2000 and is relatively stable since then (4). A similar trend has been reported in the National Health and Nutrition Examination Surveys (NHANES) with a prevalence of hypertension of 65.6% in subjects aged ≥ 60 years in 2014 (5). As expected from the opposite variations in systolic and diastolic BP, isolated systolic hypertension is the most common hypertensive phenotype observed in elderly (1, 6).

In the older adult population, increased levels of BP are associated with an increased risk of cardiovascular morbidity and mortality (7, 8). Thus, at the same level of BP, the risk of stroke, heart failure, coronary heart disease, peripheral artery disease, chronic kidney disease or dementia is several folds higher in elderly than in younger hypertensive patients (7). Today, there is a strong evidence that hypertension in the elderly as well as in the very elderly must be treated and this is supported by international guidelines and a Cochrane meta-analysis (9–13). A sub-analysis of the Systolic Blood Pressure Intervention Trial (SPRINT) in elderly has recently confirmed the benefits of lowering BP in hypertensive patients older than 75 years with some potential benefits on cognitive function and white matter lesions (14–17).

In order to prevent hypertension-mediated organ damages, patients need to follow a life-long treatment based on life-style changes and antihypertensive medications. To achieve the defined BP targets, a proper adherence to medications is essential. Yet, poor adherence to therapy has been identified as a major factor limiting the benefits of antihypertensive therapies at all ages (10). As reviewed recently, a low adherence has been associated to several issues including a high CVD incidence and mortality, a higher rate of hospitalization, and high health care expenditures (18). The purpose of this review is to discuss the specific situation of elderly hypertensive patients in regards to drug adherence and persistence and their clinical consequences.

## Prevalence of Poor Medication Adherence and Persistence in Elderly Patients

Because elderly patients often share several comorbidities needing drug therapies and might suffer from potential cognitive deficits, it is commonly assumed that poor adherence is more prevalent and more severe in elderly than in younger patients (19). Is this really the case? The prevalence of poor adherence among unselected treated hypertensive patients ranges between 20 and 30% when assessed using self-reports, medication possession ratios or pill count, which tend to overestimate the true adherence (20, 21). Interestingly, difference in adherence levels were observed between young-old (age 65–74 y) and very old patients (age >75 y), the former being more adherent than the latter (22). Long-term persistence to cardiovascular therapies including antihypertensive therapy is low in most hypertensive patients including in elderly patients (23, 24). Thus, in a British survey involving 37'643 patients with hypertension receiving a relevant medication, drug persistence at 6 months ranged between 40 and 50% (25). In a large Swedish cohort of 5,225 patients followed by 48 Swedish primary healthcare centers, persistence to antihypertensive therapy was about 70% at 2 years in hypertensive patients older than 60 y and actually significantly higher than in patients aged 30 to 49 y (26). In a Spanish survey using questionnaires addressed to patients with chronic diseases, adherent patients were older than non-adherent patients (27). In a cross-sectional study included 1,043 community-dwelling Hispanic adults with hypertension living in New York, the prevalence of high adherence was significantly better in older adults than in younger hypertensive (34 vs. 24.5%), and in older participants, age was a positive determinant of good adherence (28). In one smaller study conducted in family practices, drug adherence was found to be lower in patients aged >65 years when compared to those aged 55 to 64 years (29). Whether this difference is due to the different setting is unknown. Yet most published studies would support the hypothesis that adherence to hypertensive medication is rather better in elderly patients than in younger one, except perhaps for very elderly with cognitive deficits as will be discussed below (30). Several surveys have shown differences in adherence and persistence according to the antihypertensive drug classes, the persistence being better with blockers of the renin-angiotensin system than with calcium channel blockers, beta-blockers or diuretics (26, 31, 32). In this context, age is a major determinant of persistence. However, very few studies have examined drug persistence to the various antihypertensive drug classes according to age. In a survey performed in Ontario, Canada, between 1999 and 2010 among hypertensive patients aged 66 years or more, angiotensin-converting enzyme inhibitors, angiotensin receptor blockers and calcium channel blockers had also a better persistence than beta-blockers and diuretics (33). However, there was no additional information on drug persistence according to decades of age between 66 and 86 years for example.

At this stage however, it must be emphasized that evaluating adherence to medication adequately in elderly population is difficult and imprecise. Most studies have used either the Morisky questionnaire, pill count, interviews or pharmacy databases enabling to calculate the medication possession ratio or the percentage of days covered by the treatment. All of these methodological approaches have limitations and most of them tend to overestimate the levels of adherence (18, 34). A 4-item Krousel-Wood Medication Adherence Scale (K-Wood-MAS-4) has been developed to assess adherence using the percentage of days covered by the therapy specifically in elderly patients (35). Recent data with this tool have shown a good prediction of uncontrolled hypertension and incident cardiovascular events. In the absence of standardization of methods, it remains difficult to conclude. In addition, one has to consider that geographic factors, sometimes within a country, can modulate the association between age and adherence under the influence of local traditions, the environment and healthcare systems supporting the healthcare management of elderly patients. These factors were evaluated in many American counties by Han et al. (36) who reported that residing in medically underserved areas, counties with high deprivation scores, and not receiving Part D low-income subsidy were associated with poor adherence to antihypertensive medications.

**Figure 1:**
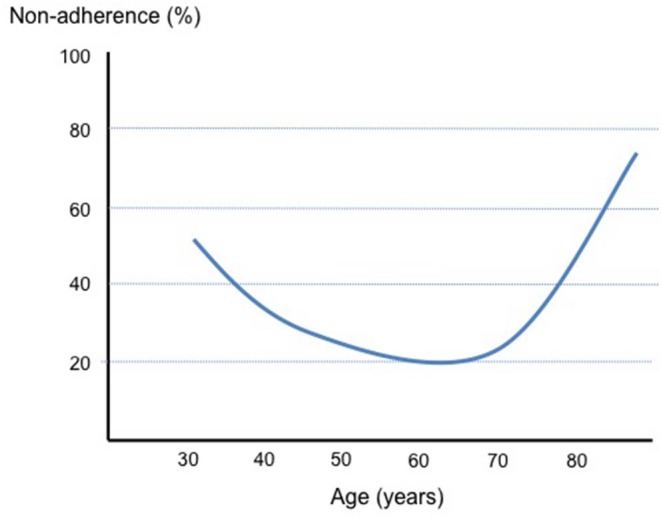
Schematic representation of the changes in the percentage of non-adherence to drug therapies according to age.

Thus, a poor adherence to hypertensive medications is common in the general hypertensive population and this is true for elderly patients as well as young hypertensive subjects. However, if anything, older age is rather a determinant of good adherence and the relationship between age and poor adherence may be U-shaped rather than linear as hypothetically illustrated in Figure 1.

## Determinants of Poor Adherence in Elderly

Elderly and younger hypertensive patients share many of the risk factors and determinants of poor adherence (34). Thus, a complex treatment regimen, a dosing frequency greater than twice a day, the fear of or the presence of side effects are well-recognized factors having an important negative impact on long-term drug adherence and persistence as well as on the risk of hospitalization (37). Yet, some risk factors have a greater impact in elderly because of the overall health context. Thus, the pill burden is often greater in older patients because of the development of comorbidities associated with aging (38). In a prospective cohort study conducted in Sweden using register data with national coverage (1,742,336 individuals aged ≥65 years), individuals were exposed to 4.6 (*SD* = 4.0) drugs on average and the prevalence of polypharmacy (5+ drugs) was 44.0%, and of excessive polypharmacy (10+ drugs) 11.7% (39).

In very old patients, beliefs that medications will not help them and will not modify their outcome may be more prominent (40). This may be a reason why many elderly patients may consciously choose not to fill a prescription or to discontinue therapy. In addition, disabilities, such as hearing or visual loss and loss of dexterity due to severe hand arthritis, may limit the capacity of elderly to handle their medications correctly. In some countries, the affordability of drug treatments represents another major limitation to an adequate adherence and persistence (41). Thus, in a survey of a large patient population, cost-related non-adherence was a significant reason of deciding not to fill or refill a prescription or skipping doses and taking smaller doses to make the medicine last longer (42). In this survey, the majority of patients was older than 65 years and the negative impact on poor drug adherence was estimated to range between 7.5 and 11%.

## Depression, Hypertension and Adherence in the Elderly

An elevated blood pressure is not only a known risk factor for cardiovascular events but it also increases the risk of incident depression in elderly (43). Thus, hypertensive older subjects showed a 37–46% increased likelihood of developing depression as compared to normotensive age-matched group in a European survey (44). *Per se* depression increases the likelihood of having functional disability or cognitive impairment 2- to 3-fold (45). In addition, symptoms of depression are strongly associated with a poor control of blood pressure in hypertension and with the development of hypertension-mediated complications (46). In a cross-sectional study of 940 patients with stable coronary heart disease (CHD), twice as many depressed participants as non-depressed participants (18 vs. 9%) reported forgetting to take their medications (47). Moreover, several studies have reported an association between depressive symptoms and a low adherence to drug therapy in hypertension (48–55). Of note, psychosocial and social frailties are also important factors leading to a poor adherence to drug therapy in elderly (56). Interestingly, in hypertensive patients with depressive symptoms, the relative risk of clinical inertia, defined as a lack of medication intensification, hypertension specialist referral, or workup for identifiable hypertension despite uncontrolled BP, was significantly higher (adjusted relative risk of 1.49; 95%CI, 1.06–2.10; *P* = 0.02) (57). Thus, for physicians and healthcare providers dealing with elderly hypertensive patients it is important to recognize depressive symptoms as they represent significant barriers to drug adherence and hence adequate blood pressure control (53).

## Cognitive Dysfunction, Adherence and Hypertension Control in Elderly

It is not well-established that elevated blood pressure and cognitive impairment (58) as well as Alzheimer disease (59) are linked and that hypertension have harmful effects on cerebral functions including cognition (60). As reviewed recently (61), there is a strong evidence that hypertension is associated with a steeper cognitive decline and poor cognitive performance and dementia and this, independently of the occurrence of stroke. Mid-life hypertension may confer a greater risk of cognitive decline than late-life hypertension (62). In older adults, the situation may differ as the relationship between blood pressure and cognitive decline may actually be U shaped. Indeed, a large analysis of two European studies has shown that hypertension might be protective in late life and that very elderly hypertensive patients with a low blood pressure might have a worse outcome, but only when they were taking antihypertensive medications (63). Similarly, in the BRONX Aging Study, the risk of dementia was particularly high in patients older than 75 y with a low diastolic blood pressure (64). Moreover, orthostatic hypotension, which occurs frequently in elderly hypertensives, is associated with a 54% higher risk of cognitive decline and dementia (65).

Cognitive dysfunction is an important determinant of poor adherence to medications because it impairs abilities in planning, organizing, and executing medication management tasks (19, 66). In addition, adequate cognitive functions are necessary to obtain medications, to follow the time schedules, to adjust doses if necessary and to deal with missed doses. In a systematic review Smith et al. (67) have recently evaluated the relationships between non-adherence and specific cognitive domains in persons with cognitive impairment, and assessed the determinants of medication non-adherence in this population has compared to subjects or patients with cognitive problems. As expected, adherence was worst in patients with some cognitive decline (ranging between 17 and 34%) as compared to controls, but this could be significantly improved when an informal caregiver was taking care of drugs administration. Executive abilities and memory were two important factors affecting adherence. The analysis also emphasized the importance of verbal memory and of accompanying caregivers such as spouse or husband at home (67). Cho et al. (68) have examined the association between cognitive function and antihypertensive medication adherence among elderly hypertensive patients using the Korean National Health Insurance Service National Sample Cohort Data of the Elderly Cohort. In this study, 20,071 elderly hypertensive patients were enrolled and patients with dementia were excluded. The prevalence of poor medication adherence to antihypertensive medications was 16.4%. In the multivariate logistic regression analysis, lower cognitive function was weakly but significantly associated with poor medication adherence (adjusted odds ratio 0.980, 95% confidence interval 0.961–0.999) (68). After a stroke event, cognitive decline is particularly frequent as illustrated in a recent survey of 108 stroke survivors (69). Indeed, in this patients' group, the prevalence of cognitive impairment at 5 years was 35.6%, and the prevalence of non-adherence ranged from 15.1% for lipid-lowering agents to 30.2% for antithrombotic.

An impaired cognitive impact may induce several forms of poor adherence in elderly patients. Indeed, frequent forms of non-adherence in the elderly include overuse and abuse of drugs, sometimes because of memory problems, forgetting, and alteration of schedules and doses (70). However, it may also be an inappropriate interruption of drug treatments, the use of medications that were prescribed for others or the ingestion of drugs that have not been prescribed by their physicians.

These studies actually confirm the conclusions of a former systematic evidence-based review conducted to identify barriers to medication adherence in cognitively impaired older adults (71). In this analysis, the main barriers to a good adherence were the ability to understand new directions, living alone, the ability to schedule medication administration into the daily routine, the use of potentially inappropriate medications, and uncooperative patients. The results of the reviews also emphasize the need to test the elderly's functional ability to manage their medication. Indeed, even small decline in cognitive decline can already affect drug adherence. This was well-demonstrate in a study by Hayes et al. who investigate the impact of very mild decline in cognitive function in 38 subjects with a mean age of 82 years (72). These subjects were living independently in the community and were asked to follow a twice-daily vitamin C regimen for 5 weeks, adherence being measured using an electronic 7-day pillbox. All of them had a normal Mini-Mental State Examination score but 18 of them had an Alzheimer's Disease Assessment Scale–Cognitive Subtest score demonstrating a mild cognitive dysfunction. At 5 weeks, adherence to vitamin C was significantly lower in the lower cognitive function group (63.9 ± 11.2% in the low vs. 86.8 ± 4.3% in the high cognitive function group, *p* = 0.007). This difference persisted after several corrections for differences in other treatment regimens. These data indicated that even a mild cognitive dysfunction has a negative impact on drug adherence, thus reinforcing the need to assess cognitive function in all very old patients. To test cognitive function, several validated tools have been proposed, which have been reviewed by Advinha et al. (73). The tool called DRUGS was the most widely used assessment instrument in the screened studies (74). DRUGS was developed as a step-wise progression of four tasks using the real regimen taken by the elderly patient: (1) identification: showing the appropriate medications, (2) access: opening the appropriate containers, (3) dosage: dispensing the correct number per dose, and (4) timing: demonstrating the appropriate timing of doses.

## Inappropriate Drug Prescribing and Adherence in Elderly

As mentioned earlier in this review, most elderly hypertensive patients suffer from concomitant diseases, which exposed them to the prescription to multiple drug therapies, frequently given by several different physicians. Thus, the probability of potentially inappropriate medications (PIM), defined as “*medications in which harm potentially outweighs the benefits, namely those that are not indicated or lack evidence of efficacy and those that do not align with patients goals/preferences and values*” is very high (75, 76). In a survey of studies published before 2012, the median rate of inappropriate medication prescriptions in primary practice was 20.5% (77). In Quebec, a retrospective population-based cohort study was conducted on more than 1 million individuals older than 66 years. In this survey, the prevalence of PIM was 48.3% (78). In Lithuania, a similar study performed on almost half a million subjects revealed that the prevalence of PIM use ranged from 24.1 to 57.2% depending on criteria used to defined PIM (79). In a more recent national survey in Portugal looking at PIM in 705 older patients followed in primary care, a potentially inappropriate medication was present in 68.6 and 46.1% of the sample had two or more (80). The impact of PIM in older as well as in younger patients is multiple. First, these medications increase the pill burden and thereby affect negatively drug adherence. Second, inappropriate drugs may interfere pharmacologically with the other prescribed treatments and may either enhance the risk of adverse reaction or decrease the efficacy of necessary drugs. This is the case for example of non-steroidal anti-inflammatory drugs (NSAIDs), which are known to increase blood pressure and to blunt the antihypertensive efficacy of diuretics or blockers of the renin-angiotensin system. In the Portuguese survey, NSAIDs belong to the top three PIM identified in elderly patients (80). Moreover, studies have suggested that PIM increases morbidity and mortality and health care costs in elderly (79, 81, 82). In all this studies, major risk factors for the prescription of PIM were being a woman, polypharmacy, multi-morbidity and mental problems.

## Deprescription and Discontinuation of Drug Therapy

Considering the high prevalence of inappropriate drug prescriptions in elderly and the major impact of these prescriptions on the occurrence of serious adverse events including mortality, the concept of deprescription has been elaborated in order to prevent further patients' harms (83). According to this concept, deprescribing is the process of tapering or stopping drugs, aimed at minimizing polypharmacy and improving patient outcomes. Scott et al. actually proposed a 5-step deprescribing protocol as summarized in Table 1.

**Table 1:** The five steps of the deprescribing process.

(1) To ascertain all drugs the patient is currently taking and the reasons for each one
(2) To consider overall risk of drug-induced harm in individual patients in determining the required intensity of deprescribing intervention
(3) To assess each drug in regards to its current or future benefit potential compared with current or future harm or burden potential
(4) To prioritize drugs for discontinuation that have the lowest benefit-harm ratio and lowest likelihood of adverse withdrawal reactions or disease rebound syndromes
(5) To implement a discontinuation regimen and monitor patients closely for improvement in outcomes or onset of adverse effects

*The full protocol can be viewed in the original paper by Scott et al. (83)*.

In a systematic review of withdrawal trials including stopping antihypertensive agents, it has been demonstrated that in patients 65 years and older, some drugs could be discontinued without harm in between 20 and 100% of patients, provided patients were adequately selected and monitored closely after the withdrawal (84). In the Australian National Blood Pressure study, 37% of participants remained normotensive 1 year after drug withdrawal (85). Withdrawing inappropriate antihypertensive agents was also associated with fewer cardiovascular events and deaths over a 5-year follow-up period (86). Deprescribing drugs has also been shown to be effective in older patients with chronic kidney disease who have a very high pill burden (87). However, it is not always easy to select hypertensive patients in whom a drug withdrawal or a drug reduction could be beneficial without increasing the risk of a cardiovascular event. Thus, when patients have suffered from symptomatic orthostatic hypotension or have experienced several unexplained falls, a reduction of the antihypertensive therapy appears to be obvious. However, the situation is not always as clear. In a prospective randomized study conducted in the community, Dutch investigators have reported that treatment discontinuation in patients 75 years or older with mild cognitive dysfunction did not result in an improvement of cognitive and psychological functions at 16 weeks, despite the fact that BP increase by a mean of about 7/2.5 mmHg (88). However, the same authors found a significant improvement in orthostatic hypotension in the patients' group in which drug therapy was discontinued (89). In a Brazilian study, older subjects (>60 years of age) who discontinue the use of their antihypertensive treatment had a 3-fold higher risk of cardiovascular mortality than those continuing treatment after 11 years of follow-up (90). Thus, withdrawing antihypertensive drug therapies in elderly might not always be innocent depending on the level of cardiovascular risk of the patient. The fear of an event is one reason why physicians are often reluctant and uncomfortable changing antihypertensive medications even in advanced age (91).

## Risk of Non-Adherence in Elderly

Partial or total non-adherence to antihypertensive therapy is associated with an increased risk of cardiovascular event and death at all age (18). However, the risk is proportionally higher in old patients because they often suffer from multiple comorbidities and have intrinsically a higher cardiovascular risk. Thus, in a population-based cohort study of Medicare beneficiaries aged 66–79 years who were newly diagnosed with hypertension and initiated on antihypertensive in 2008–2009 (*n* = 155, 597), the incidence of cardiovascular events (myocardial infarction, ischemic heart disease, stroke/TIA, congestive heart failure) was 2-fold higher in those subjects whose treatment covered <80% of the days. This situation concerned about 40% of the studied population (92). Studies have demonstrated that a poor adherence increases the incidence of hospitalization in elderly as well as the risk of death (19). It is therefore mandatory to find solutions to prevent non-adherence in older hypertensive patients. Among the proposed strategies, it is recommended: (1) to carefully assess the need for antihypertensive medications; (2) to increase the use of single pill combinations in order to reduce the pill burden; (3) to favor the use of long-acting drugs covering for missed doses; (4) to support patients either with members of the family or friends or with reminders or pill organizers prepared for example by pharmacists (10).

## Conclusions

Drug adherence is a crucial issue in the pharmacotherapy of chronic diseases at all ages. However, in contrast to the general believe drug adherence is better rather than lower in patients aged 65–80 years when compared to young adults (<50 years). Yet, in very old patients, adherence to medications tend to decrease for many reasons, one of them being the progressive cognitive decline or depression developing with age. In order to avoid frequent and costly hospitalizations, physicians should periodically reassess the pertinence of all prescribed medications including those prescribed to lower blood pressure, in order to prevent potentially inadequate medications. In addition, elderly patients may benefit from supports provided by other health care providers, who are closer to their home such as pharmacists (93) or visiting nurses in the context of integrated care system (94). In recent years, even follow-up by non-medical professionals such as barbers in the US or hairdressers in Europe have been shown to offer a feasible and valid alternative for the screening and follow-up of hypertension (95, 96). Such approaches should improve the quality of life of elderly hypertensive and help containing health care costs.

## Author Contributions

MB has written the first draft. EP and GW have read and revised the manuscript and contributed to references search.

### Conflict of Interest

The authors declare that the research was conducted in the absence of any commercial or financial relationships that could be construed as a potential conflict of interest.
